# Estimating the Numbers of Malaria Infections in Blood Samples Using High-Resolution Genotyping Data

**DOI:** 10.1371/journal.pone.0042496

**Published:** 2012-08-29

**Authors:** Amanda Ross, Cristian Koepfli, Xiaohong Li, Sonja Schoepflin, Peter Siba, Ivo Mueller, Ingrid Felger, Thomas Smith

**Affiliations:** 1 Swiss Tropical and Public Health Institute, Basel, Switzerland; 2 University of Basel, Basel, Switzerland; 3 Infection and Immunity Division, Walter and Eliza Hall Institute, Parkville, Australia; 4 BG Medicine Inc., Waltham, Massachusetts, United States of America; 5 Papua New Guinea Institute of Medical Research, Goroka, Eastern Highlands Province, Papua New Guinea; 6 Barcelona Centre for International Health Research, Barcelona, Spain; Instituto de Higiene e Medicina Tropical, Portugal

## Abstract

People living in endemic areas often habour several malaria infections at once. High-resolution genotyping can distinguish between infections by detecting the presence of different alleles at a polymorphic locus. However the number of infections may not be accurately counted since parasites from multiple infections may carry the same allele. We use simulation to determine the circumstances under which the number of observed genotypes are likely to be substantially less than the number of infections present and investigate the performance of two methods for estimating the numbers of infections from high-resolution genotyping data.

The simulations suggest that the problem is not substantial in most datasets: the disparity between the mean numbers of infections and of observed genotypes was small when there was 20 or more alleles, 20 or more blood samples, a mean number of infections of 6 or less and where the frequency of the most common allele was no greater than 20%. The issue of multiple infections carrying the same allele is unlikely to be a major component of the errors in PCR-based genotyping.

Simulations also showed that, with heterogeneity in allele frequencies, the observed frequencies are not a good approximation of the true allele frequencies. The first method that we proposed to estimate the numbers of infections assumes that they are a good approximation and hence did poorly in the presence of heterogeneity. In contrast, the second method by Li *et al* estimates both the numbers of infections and the true allele frequencies simultaneously and produced accurate estimates of the mean number of infections.

## Introduction

People who live in malaria-endemic areas may have several concurrent infections. Accurately discriminating between these both yields the multiplicity of infection (MOI), an epidemiological measure of the number of infections per individual, and can improve the understanding of many areas of malariology, such as the dynamics of infections, pathogenesis, effect of transmission intensity, drug efficacy and parasite genetics.

*Plasmodium* populations are highly diverse. Polymerase chain reaction (PCR)-based genotyping using polymorphic loci has been established to discriminate parasite clones within an individual. Whilst PCR can detect the alleles of parasites present in a blood sample, it does not always give an accurate count of the infections present since parasites from multiple infections may bear the same allele or alleles close in size. High-resolution techniques have increased the discriminatory power by more precisely determining the size or sequence of the alleles. However since the true number of infections is unknown, the accuracy of high-resolution techniques cannot be determined. Two questions arise: (i) Is there substantial underestimation of the multiplicity in the blood samples due to multiple indistinguishable genotypes? (ii) What is the distribution of the number of infections in the population from which the sample was drawn?

There have been few attempts to address these issues. Carter and Mcgregor [1] derived a formula to estimate the mean number of infections using data on a single locus which has two alleles. Hill and Babiker [2] extended the equations to incorporate multiple alleles and loci. However with the large number of alleles distinguished using high-resolution genotyping, this method becomes cumbersome to implement. Li and colleagues developed models which have the principal aim of estimating either haplotype frequencies [3] or haplotype-trait associations [4], but can also allow the number of infections to be estimated.

There is a lack of information on the circumstances under which the number of infections and number of observed genotypes differ substantially. Factors such as the number of alleles, the heterogeneity of allele frequencies, number of blood samples and mean number of infections are likely to play a role.

In this paper, we (a) present simulations to evaluate the impact of different factors on the disparity between the number of observed genotypes and the number of infections present and (b) evaluate two methods of estimating the numbers of infections.

## Methods

Due to the large number of alleles distinguished by high-resolution genotyping, we focus on one marker gene. Adding information from a second marker is not justified since in practice it would not greatly enhance the ability to distinguish between infections and would increase complexity.

### Simulations to determine when the numbers of infections and observed genotypes differ substantially

For each individual simulated blood sample, we randomly generated the number of infections and then randomly selected an allele for each infection. We then determined the numbers of observed genotypes. The simulations only refer to the time that a blood sample is taken, giving a cross-sectional snapshot of the infections present in an individual. We do not simulate the processes of acquiring and clearing infections.

We defined a baseline scenario with 100 blood samples, the number of infections per blood sample following a zero-truncated Poisson distribution with a mean of 5.03 infections corresponding to 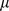 of 5, and 20 alleles of equal frequency. We investigated the effect of the different factors by varying them one by one (Table 1). A simulated sample of 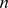 blood samples will have stochasticity due to the random generation of both the numbers of infections per blood samples from a distribution and the alleles assigned to each infection. We captured the variability in the mean number of observed genotypes by simulating 101 sets for each scenario, each set with 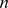 blood samples. The minimum and maximum mean from the 101 sets are presented.

**Table 1:** Factors investigated by the simulations.

parameter	values*
number of alleles†	2, 3, 4, 5, 7, 15, **20**, 30, 50, 100
frequency of most common allele‡	**0.05**, 0.1, 0.2, 0.3, 0.4, 0.5, 0.6, 0.7, 0.8, 0.9
mean number of infections	1.13, 1.27, 1.58, 2.31, 3.16, 4.07, **5.03**, 6.01, 7.01, 8.00, 9.00, 10.00, 11.00
distribution of numbers of blood samples	**zero-truncated Poisson**, zero-truncated negative binomial
number of blood samples	5, 10, 15, 20, 30, 50, **100**

* The baseline scenario is indicated by bold font.

† All alleles have equal frequency.

‡ The remaining alleles have equal frequency.

### Methods to estimate the numbers of infections from high-resolution genotyping data

We present two potential methods, one proposed here and one previously described. They differ in their approach but, in practice, the most important difference between them is that the first estimates the numbers of infections assuming that the observed allele frequencies are a good approximation of the true allele frequencies, whereas the second is able to estimate numbers of infections and true allele frequencies simultaneously. Thus we investigate whether the added complexity of the second method is necessary to provide accurate estimates.

#### Method 1: Estimating numbers of infections only

The first model estimates the parameters of the distribution of the numbers of infections. The observed allele frequencies are used to derive the conditional probabilities of observing 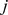 genotypes given that there are 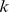 infections present in a blood sample. These conditional probabilities are then combined in a Bayesian model with the frequencies of observed genotypes and an assumed distribution for the numbers of infections. The details are provided below. This model does not estimate the true allele frequencies but assumes that the allele frequencies are either known from another source or that the observed allele frequencies are a good approximation of the true allele frequencies.

#### Details of Method 1

We are interested in the unknown frequencies of 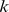 infections in the selected blood samples, 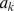, and in the population from which they were drawn, 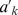 for 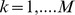. We choose the integer 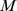 to be greater than the maximum number of observed genotypes with the intention that 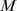 is larger than the unknown maximum number of infections per blood sample.

The observed frequencies of blood samples with 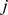 genotypes, 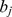, follow a multinomial distribution 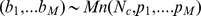 where 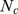 is the number of blood samples in a population survey and 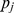 is the probability of a blood sample having 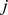 observed genotypes.

We derive 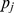 using the conditional probabilities of observing 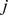 genotypes given that 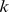 infections are present, 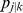


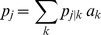
We obtain 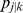 from the observed allele frequencies using simulation. This avoids the need to determine the equations, which become complicated for large 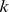. For each 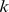, we randomly select 100,000 samples of 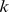 alleles and count the frequencies of the numbers of distinct genotypes. The true allele frequencies are unknown, we assume that the observed allele frequencies are a reasonable approximation and that the alleles are randomly distributed.

We allow for sampling variation in the numbers of infections. We specify that the frequencies of infections in the sample of blood samples, 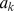, arise from a multinomial distribution 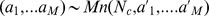 where 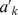 are the population frequencies we wish to estimate. We assume that 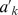 are specified by the probability function of a chosen distribution and estimate the values of the parameters describing this distribution to obtain our estimates.

The model was fitted using the Metropolis-Hastings algorithm in WinBUGS [5]. We used R to simulate the conditional probabilities, pass the inputs to WinBUGS via R2Bugs [6] and return the results.

#### Method 2: Estimating both numbers of infections and allele frequencies simultaneously

The second method estimates both the distribution of the numbers of infections and the true allele frequencies simultaneously using the Expectation-Maximization algorithm [7]. The model is described by Li *et al* [3] and is implemented in R [6] using the package malaria.em. This model was originally aimed at estimating multi-loci haplotype frequencies, a more complicated problem, but is readily used here with data from only one marker gene. By iteratively alternating between performing an expectation (E) step, which computes the expectation of the log-likelihood evaluated using the current estimate for the parameters, and a maximization (M) step, which computes parameters maximizing the expected log-likelihood found on the E step, the EM approach finds the maximum likelihood estimates of the parameters. The numbers of infections are assumed to follow a zero-truncated Poisson distribution.

To evaluate how well these methods perform in different circumstances, we applied them to the simulations described above. We also tested how well they performed when the assumption of a Poisson distribution for the numbers of infections is incorrect by applying them simulated numbers of observed genotypes generated from negative binomial distributions with different variances.

### Example using genotyping data

We apply both methods to data from a study of malaria infections in children in Maprik District, Papua New Guinea [8]–[11]. *Plasmodium falciparum* and *P. vivax* are both present in this area. Children aged one to three years at enrolment were followed up over 16 months, but only blood samples from the baseline survey were included in this analysis. There were 190 children enrolled in March 2006, but only samples positive by microscopy or LDR, a molecular method for Plasmodium species detection [12], were genotyped using high-resolution capillary electrophoresis-based PCR. The *P falciparum* infections were genotyped using one marker *(msp2)* and the *P vivax* infections with two markers (*msp1*F3 and MS16).

### Ethics statement

The cohort study was approved by institutional review boards of the PNG Medical Research Advisory Committee (approvals 05.19 and 09.24), University Hospitals Case Medical Center (Cleveland, Ohio USA), and the Ethikkommission beider Basel (approval 03/06). Informed written consent was provided by the parents or legal guardians of each child.

## Results

### Simulations to determine when the numbers of infections and observed numbers of genotypes differ

**Figure 1:**
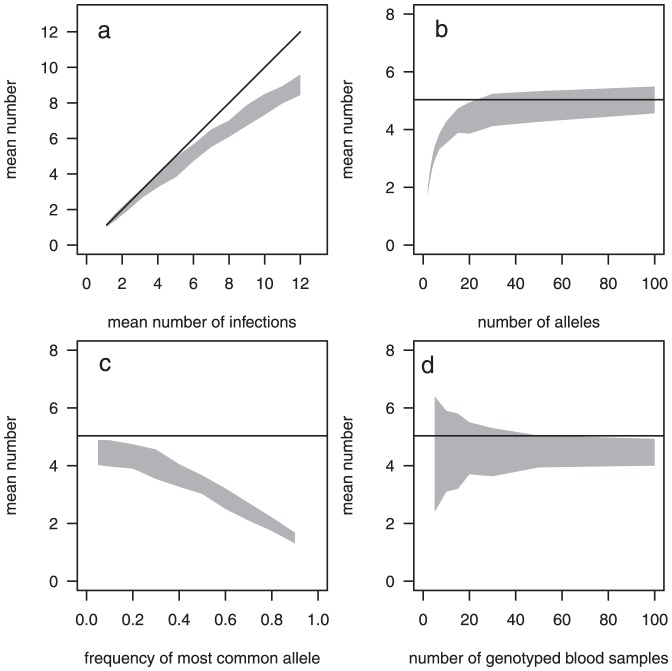
Impact of factors on the simulated mean number of genotypes. a: Mean number of infections b: number of alleles c: frequency of most common allele d: number of blood samples. Solid line: mean number of infections, shaded polygon: minimum and maximum of mean observed number of genotypes for the 101 simulated sets of 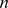 blood samples. Baseline case: 100 children, 20 alleles of equal frequency, the number of infections per blood sample follows a zero-truncated Poisson distribution with a mean of 5.03 (Table 1). Each panel shows the effect of varying one factor from the baseline case.

There was variation in the simulated mean numbers of observed genotypes due to random variation in numbers of infections and in the alleles selected (Figure 1, shaded polygons). The disparity between the number of infections and number of observed genotypes was greater with increasing mean numbers of infections (Figure 1a), decreasing numbers of alleles (Figure 1b) and increasing heterogeneity in allele frequencies (Figure 1c). Small sample sizes lead to increased variability but there was no substantial difference between the true number of infections and the numbers of genotypes observed (Figure 1d). The disparity was small where there were 20 or more alleles, 20 or more blood samples, a mean MOI of 6 or less and where the frequency of the most common allele was no greater than 20%.

### Evaluation of methods to estimate the numbers of infections

Both methods were applied to the simulated data above to evaluate how well they could reproduce the mean number of infections. The methods were applied to each of the 101 sets of 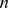 simulated blood samples for each scenario and the distributions of estimated means are shown as boxplots.

**Figure 2:**
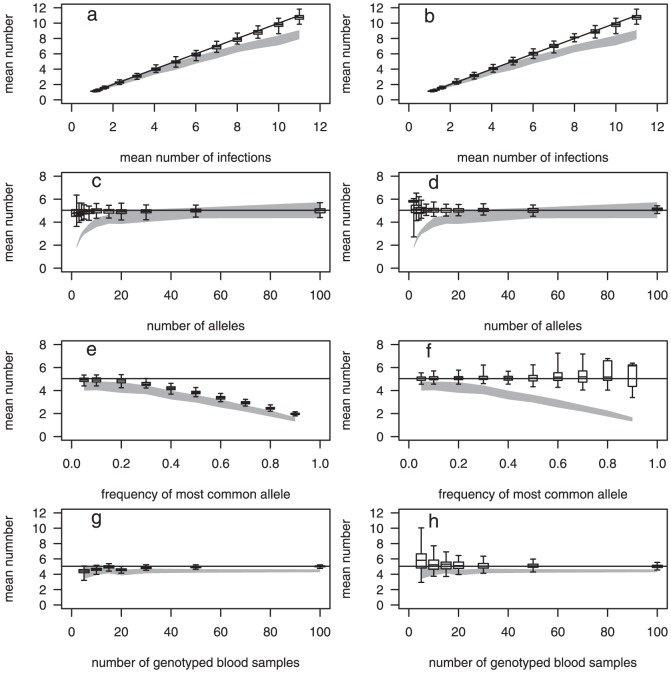
Model performance: estimated numbers of infections from the simulated sets of blood samples. LH column: Method 1, RH column: Method 2. Solid line: true mean number of infections, shaded polygon: minimum and maximum of mean observed number of genotypes for the 101 sets (samples of 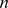 blood samples) for each scenario. Boxplots: Estimates of the mean number of infections for each of the 101 sets. Baseline scenario: 100 children, 20 alleles of equal frequency, the number of infections per blood sample follows a zero-truncated Poisson distribution and a mean of 5.03 (Table 1).

**Figure 3:**
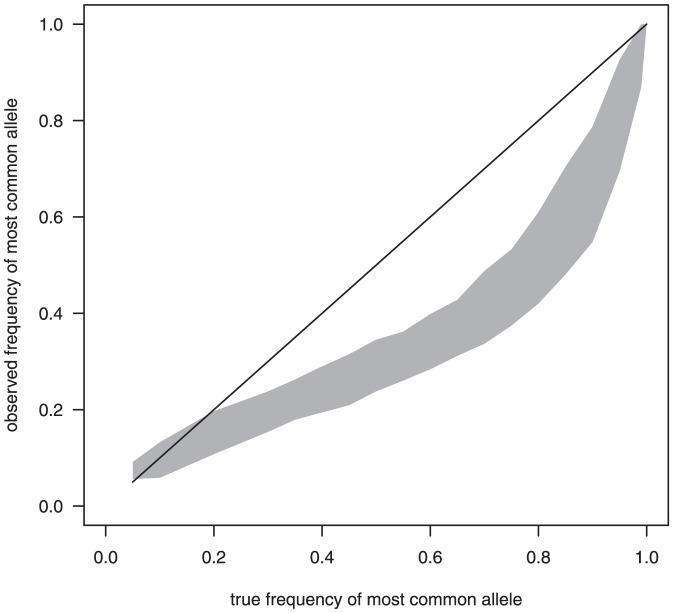
Actual and observed frequency of the most common allele. Solid line: line of equality, shaded polygon: the minimum and maximum observed frequencies of the most common allele for the 101 sets (each of 100 blood samples) for each scenario. There were 20 alleles, the frequency of the most dominant allele is shown on the X-axis, the remaining 19 had equal frequency. The number of infections per blood sample followed a zero-truncated Poisson distribution with a mean of 5.03.

The estimates using Method 1 (Figure 2, left hand column) were generally good where the observed allele frequencies would be a reasonable approximation of the true allele frequencies. Where there was heterogeneity in allele frequencies, however, they were poor (Figure 2e). The observed allele frequencies are a poor approximation of the true frequencies in the presence of heterogeneity in the allele frequencies (Figure 3). This is because the chance of multiple infections with the same allele occurring in the same blood sample is greater the higher the allele frequency. If the true allele frequencies were known, then Method 1 performed well (results not shown).

The second method by Li *et al* (Figure 2, right hand column) provided unbiased estimates even if there was heterogeneity in allele frequencies. The variability of the estimates was large under circumstances where there was very little information such as for a very large degree of heterogeneity in allele frequencies, small numbers of blood samples or very few alleles. Coverage of the 95% confidence intervals was reasonably good for both methods (not shown), ranging from 91% to 98%, when restricted to circumstances when the bias was low. However coverage fell dramatically when there was substantial heterogeneity in allele frequencies with Method 1.

**Figure 4:**
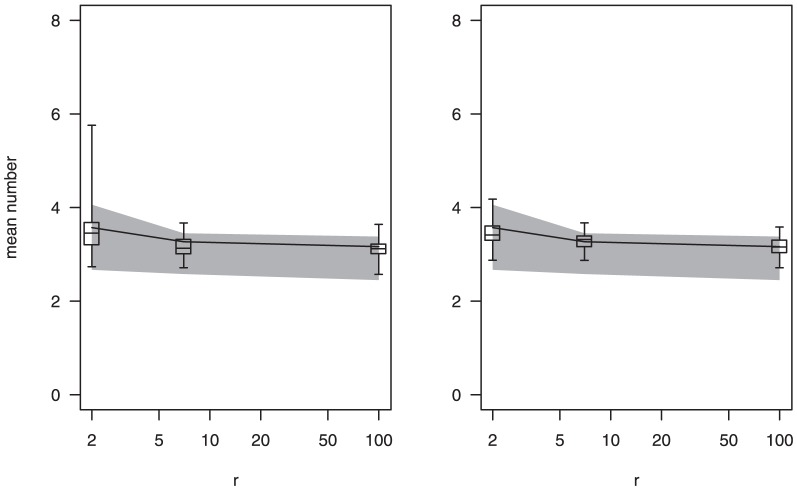
Model performance when the assumed distribution for the numbers of infections is incorrect. The observed numbers of genotypes were simulated using numbers of infections following negative binomial distributions, however the models assume that the numbers of infections follow a Poisson distribution. The parameter 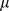 was set to 3. A small value of 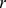 indicates larger variation and skew compared to a Poisson distribution whereas a value of 100 is similar to a Poisson. LH: Method 1 RH: Method 2. Solid line: true mean number of infections, shaded polygon: minimum and maximum of mean observed number of genotypes for the 101 sets (each set of 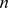 blood samples) for each scenario. Boxplots: Estimates of the mean number of infections for each of the 101 sets. We simulated blood samples from 100 children and 20 alleles of equal frequency (Table 1).

The mean number of infections was reasonably accurately estimated even when the assumption of a Poisson distribution for the numbers of infections was incorrect, unless the extra-Poisson variation was large (Figure 4). However, although we tested this assumption using the negative binomial distribution, we did not exhaustively test all possible distributions.

### Application to data

Of the samples from 190 children aged 1 to 3 years in Maprik district, Papua New Guinea, 76 (40%) were positive by microscopy or LDR for *P falciparum* and 80 (42%) for *P vivax*. The number of alleles observed ranged from 31 to 67 for the 3 markers (Table 2). There was little difference between the mean number of observed genotypes and estimated mean number of infections in this dataset suggesting that there were few infections obscured by shared alleles. Both methods estimated slightly a different mean MOI for *P vivax* infections from the two markers. This is likely to be due to the slightly lower detectibility of MS16 compared to *msp1*F3 [10].

**Table 2:** Data summary and estimated mean numbers of infections.

parameter	*P. falciparum msp2*	*P. vivax MS16*	*P. vivax msp1*F3
number of blood samples included	76	80	79
number of alleles detected	33	67	31
frequency of most common allele	15%	5%	24%
mean number of observed genotypes	1.71 (1.47, 1.95)	2.19 (1.89, 2.49)	2.21 (1.90, 2.53)
maximum number of observed genotypes	7	6	6
heterozygosity‡ 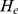	0.93	0.98	0.87
Estimated mean number of infections:			
method 1	1.87 (1.57, 2.22)	2.26 (1.89, 2.80)	2.58 (2.07, 3.28)
method 2	1.80 (1.41, 2.23)	2.26 (1.70, 2.89)	2.54 (2.23, 2.86)

‡ The expected heterozygosity 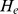 is the probability that 2 clones taken at random from the population carry different alleles.

## Discussion

Our simulations indicate that the problem of indistinguishable alleles is unlikely to lead to substantial underestimation of the number of infections by using the number of observed genotypes. The size of the disparity was small for 20 or more alleles, a sample size of 20 or greater, a mean MOI of 6 or less and where the frequency of the most common allele was no greater than 20%. We did not, however, simulate combinations of these variables. Since none of the baseline values we adopted (100 blood samples, 20 alleles of equal frequency, a zero-truncated Poisson distribution with mean 5.03 infections) strongly impacted on the number of observed genotypes, it seems unlikely that a more generous value in one variable could compensate for poor value in another, such as a marker with poor discriminatory ability in combination with a larger number of blood samples. These approximate cut-offs could serve as criteria for selecting genetic markers for discriminating between infections in an individual. They also support guidelines for the related problem of distinguishing recrudescences from new infections in clinical trials of drug efficacy [13] and justify the use of statistical methods which assume that the observed MOI is reasonably accurate [14].

Accurate estimates of the mean number of infections could be obtained. Both methods investigated in this study differ from previous studies [1], [2] in that, facilitated by the large number of alleles detected by high-resolution genotyping, they concentrate on only one marker gene. Assumptions about the allele frequencies were found to be important. The method by Li *et al*, which uses the EM algorithm to estimate both the numbers of infections and the true allele frequencies simultaneously, provided unbiased estimates. In contrast, method 1 which assumes that the observed allele frequencies are similar to the true allele frequencies had biased estimates in some cases. This method would only be appropriate where the observed allele frequencies are similar to the true frequecies (such as for homogenous allele frequencies or low MOI) or if they are known from another source. The observed allele frequencies suffer from the same problem that multiple infections bearing the same allele are not counted. This is also true of prevalence, and the distinction between the prevalence and frequency of alleles has been previously illustrated [15].

Neither method takes detectability into account. The blood samples do not necessarily contain parasites from all the infections present if there are low densities in the blood stream. This could be due to a low parasite biomass, sequestration or synchronisation. When the parasites are present at low levels in the sample, they may not be detected due to methodological constraints with PCR [16], [17]. In multi-clonal infections, minority variants might be missed due to being outcompeted in the competition for primers or other consituents of the reaction mix [10]. There is variation in assay sensitivity between different laboratories [18] and between different markers. Reported estimates of the probability of dectecting a clone lie in the range of 0.4 to 0.9 for *P. falciparum* and *P. vivax* [10], [11], [19], [20]. The model estimates refer only to infections which would have been counted if they had been distinguished in the genotyping.

We assume that the alleles are distributed at random. In the case of markers associated with drug resistance, the distribution of alleles is unlikely to be random [21] although these are unlikely to be useful for estimating the MOI. The assumption would also be untrue if there are multiple blood samples from the same hosts, especially if they are taken within a short time period. Correlation between alleles could also arise due to sibling infections which could occur if gametocytes from multiple infections are taken up in the same blood meal and, following meiosis, multiple related sporozoites are injected into a person in one bite. Recent work has suggested that there may be discrete spatial clusters of *P falciparum* parasite types [22].

This problem is distinct from the adjustment of antimalarial drug efficacy trial results using PCR data. In this case, interest centres on whether a single infection detected following treatment signifies a new infection or a treament failure. It has been considered elsewhere [17], [23]–[28].

Method 1 does not estimate the number of infections in an individual blood sample, but rather only the distributions which best approximate the population frequencies. Method 2, by Li *et al*, can additionally give the posterior probabilities for different configurations of marker genes and numbers of infections corresponding to the observed genotypes for each individual.

Extensions could be made by including the option of a negative binomial distribution for the number of infections in a host, and by considering non-random selection of alleles. For the method we had proposed (method 1), in theory it would also be possible to estimate the allele frequencies in addition to the numbers of infections. However, in practice, simulating the matrix of conditional probabilities, 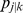, at each iteration would be very time-consuming.

In conclusion, we have shown that with high-resolution genotyping the problem of underestimating numbers of infections due to multiple infections bearing the same allele is unlikely to be substantial. Accurate estimates of the mean number of infections can be obtained with the method by Li *et al* which can be implemented in R using the package malaria.em. In most surveys of malaria infection, far more than 20 blood samples will be typed and the mean numbers of observed genotypes as detected by typing size polymorphic markers have been reported to be lower than 6 even in areas of intense transmission. Most markers established for genotyping have exhibited more than 20 alleles with frequencies usually below 20%. Under such circumstances, the difference between the mean number of infections and observed genotypes is likely to be small.
